# Pathogenic microRNA’s in myeloid malignancies

**DOI:** 10.3389/fgene.2014.00361

**Published:** 2014-11-19

**Authors:** Mona Khalaj, Montreh Tavakkoli, Alec W. Stranahan, Christopher Y. Park

**Affiliations:** ^1^Weill Graduate School of Medical Sciences, Cornell UniversityNY, USA; ^2^Human Oncology and Pathogenesis Program, Memorial Sloan Kettering Cancer CenterNY, USA; ^3^Pathology and Laboratory Medicine, Memorial Sloan Kettering Cancer CenterNY, USA

**Keywords:** microRNAs, leukemia, hematopoiesis, hematologic malignancies, leukemogensis

## Abstract

Recent studies have significantly improved our understanding of the role microRNAs (miRNAs) play in regulating normal hematopoiesis. miRNAs are critical for maintaining hematopoietic stem cell function and the development of mature progeny. Thus, perhaps it is not surprising that miRNAs serve as oncogenes and tumor suppressors in hematologic malignancies arising from hematopoietic stem and progenitor cells, such as the myeloid disorders. A number of studies have extensively documented the widespread dysregulation of miRNA expression in human acute myeloid leukemia (AML), inspiring numerous explorations of the functional role of miRNAs in myeloid leukemogenesis. While these investigations have confirmed that a large number of miRNAs exhibit altered expression in AML, only a small fraction has been confirmed as functional mediators of AML development or maintenance. Herein, we summarize the miRNAs for which strong experimental evidence supports their functional roles in AML pathogenesis. We also discuss the implications of these studies on the development of miRNA-directed therapies in AML.

## INTRODUCTION

Hematopoietic stem cells (HSCs) exhibit the unique ability to undergo self-renewal and to give rise to all cells of the hematopoietic system throughout the lifetime of an organism (Weissman, 2000; Kondo et al., 2003). In order for HSCs to maintain hematopoiesis, the balance between self-renewal and differentiation is finely regulated under both steady-state and stress conditions. Several molecular networks that control these processes have been identified in recent years (Ramalho-Santos et al., 2002; Park et al., 2003; Zhang et al., 2006; Miyamoto et al., 2007; Deneault et al., 2009; Hope et al., 2010; Aguilo et al., 2011; Ting et al., 2012). As a part of this effort, microRNAs (miRNAs) have been identified as regulators of HSC maintenance and lineage commitment (Kluiver et al., 2007; Vasilatou et al., 2010). For example, *miR-223* regulates granulopoiesis (Fazi et al., 2005), *miR-221* and *miR*-222 negatively control erythropoiesis (Felli et al., 2005), and *miR-146*, *miR-150,* and *miR-181* promote B-lymphocyte development (Chen et al., 2004; Zhou et al., 2007; Cameron et al., 2008).

Given the role of miRNAs in regulating normal hematopoiesis, it is perhaps not surprising that miRNA misexpression may contribute to the development of hematopoietic malignancies such as acute myeloid leukemia (AML). AML consists of a heterogeneous group of malignancies characterized by the accumulation of immature blasts and limited production of normal blood cell components in the bone marrow (BM). While better supportive care practices have mildly improved the prognosis of AML in the past two decades, approaches to treat AML have remained essentially unchanged. Thus, understanding the molecular mechanisms regulating the pathogenesis of AML is of great interest.

The importance of miRNAs in carcinogenesis has been inferred by their localization to genomic regions that are frequently deleted or amplified, and to their presence near translocation breakpoints, in various human cancers (Calin et al., 2004). The relevance of miRNAs to hematologic malignancies was first established when *miR-15 and miR-16* were shown to be the critical genetic elements deleted from chromosome 13q14 in a significant proportion of chronic lymphocytic leukemia (CLL) patients. In the context of AML, gene expression profiling of AML patient blasts revealed a widespread deregulation of miRNAs. These studies also established associations between different miRNA signatures and specific molecular subtypes of disease, suggesting their potential role in AML pathogenesis (Dixon-McIver et al., 2008; Garzon et al., 2008a; Jongen-Lavrencic et al., 2008). The results of these studies can be found in a number of previously published reviews (Kluiver et al., 2006; Havelange et al., 2009; Vasilatou et al., 2010; Chung et al., 2011).

While these gene expression analyses have been used to document the transcriptional dysregulation of miRNAs in AML and to identify potential diagnostic and prognostic miRNAs, they have provided limited definitive evidence regarding the roles of miRNAs in AML pathogenesis. In fact, few miRNAs have been experimentally validated as mediators of initiation and/or maintenance of AML. In this review, we will focus our discussion on miRNAs for which a functional link between miRNA dysregulation and the development of AML has been established, including miRNAs*-125, -146, -155, -142,* and -*29*.

## miR-125

*miR-125* is among one of the most extensively studied miRNAs in the hematopoietic system. Members of the *miR-125* family are located in three highly conserved miRNA clusters throughout the human genome. These clusters include *miR-125a/miR-99b/let-7e*, *miR-125b-2/miR-99a/let-7c-1*, and *miR-125b-1/miR-100/let-7a-2* located on human chromosomes 19, 21, and 11, respectively. The *miR-125* family is highly conserved across species, with the same clusters identified on chromosomes 17, 16, and 9 in the mouse genome.

## *miR-125* IN HSC SELF-RENEWAL AND SURVIVAL

A number of groups have observed high expression of *miR-125* family members in HSCs and decreased expression during myeloid differentiation, suggesting that *miR-125* positively regulates HSC function (O’Connell et al., 2010; Ooi et al., 2010; Gerrits et al., 2012). O’Connell et al. (2010) ectopically expressed *miR-125b* in human CD34+ cells and showed that this leads to a significant increase in the number of CD45+ cells and to the expansion of human stem and progenitor cells (HSPCs) in the bone marrow of xenotransplanted mice (O’Connell et al., 2010). Similarly, Ooi et al. (2010) ectopically expressed *miR-125b* at levels 35-fold higher than endogenous levels via lentiviral transduction in mouse HSCs. Upon transplantation of these transduced HSCs, recipient mice displayed increased numbers of HSCs, but not of other immature hematopoietic cells, in both primary and secondary recipients. *miR-125b* overexpression was also shown to reduce the levels of apoptosis in HSCs, a process likely mediated through *miR-125b* inhibition of the pro-apoptotic genes *Klf13* and *Bmf*. Thus, these findings suggest that *miR-125b* promotes HSC self-renewal by promoting HSC survival (Ooi et al., 2010). Consistent with this prediction, overexpression of *miR-125b* by 100-1000 fold in HSC-enriched bone marrow significantly improved engraftment in lethally irradiated recipients (Gerrits et al., 2012). Similarly, Guo et al. (2010) described an 8-fold increase in HSC number following enforced expression of *miR-125a*. Lastly, 5-fluorouracil (5-FU)-treated BM cells overexpressing *miR-125*a displayed increased HSC function as measured by day-35 cobblestone-area forming cell (CAFC) activity and long-term transplantation assays (Gerrits et al., 2012). Therefore, both *miR-125a* and *miR-125b* appear to be potent mediators of HSC self-renewal.

## *miR-125* IN HEMATOPOIESIS AND LEUKEMIA

*miR-125b* can act as a tumor suppressor or an oncogene depending upon tumor type. In breast cancer, *miR-125b* appears to act as a tumor suppressor, as high levels of *miR-125b* inhibit the expression of the proto-oncogenic proteins ERBB2 and ERBB3 (Sonoki et al., 2005; Bousquet et al., 2008). In contrast, in prostate cancer, *miR-125b* exhibits pro-oncogenic activity, with high *miR-125b* expression inducing androgen-independent growth through the negative regulation of *Bak1*, a pro-apoptotic *Bcl2* family member (Shi et al., 2007). In AML, *miR-125b* is strongly up-regulated in patient blasts, and both *in vivo* and *in vitro* models suggest that *miR-125b* can promote the transformation of normal hematopoietic cells into malignant cells.

Insight into the biological effects of *miR-125b* comes from both *in vitro* and *in vivo* ectopic expression studies. *miR-125b* overexpression blocks terminal (monocytic and granulocytic) differentiation in HL60 and NB4 AML cell lines (Bousquet et al., 2008; Klusmann et al., 2010) and confers interleukin-3 (IL-3) growth independence to the leukemic cell line, 32Dclone3 (Bousquet et al., 2012). *ABTB1* and *CBFB* have been identified as *miR-125b* targets that may mediate these anti-apoptotic and pro-proliferative effects (Lin et al., 2011; Bousquet et al., 2012). *In vivo*, primary recipients of HSCs overexpressing *miR-125b* (35-fold) display myeloid-biased differentiation and expansion at the expense of B cells, while secondary recipients develop a lymphoproliferative disease. This increase in lymphocyte output is likely due to the preferential expansion of lymphoid-biased Slam^-^ HSCs, as they display intrinsically higher basal apoptotic rates, which makes them more prone to *miR-125*’s anti-apoptotic effects. Furthermore, a 35-fold overexpression of *miR-125b* was also associated with an expansion of common lymphoid progenitors (CLPs; Ooi et al., 2010). Consistent with these studies, enforced expression of *miR-125*a in BM cells by 1000-fold enhanced long-term reconstitution of all blood lineages following transplantation. This effect persisted in secondary transplants, although it was also associated with increased myeloid cell output (Guo et al., 2010; Gerrits et al., 2012). The majority of these mice also exhibited a myeloproliferative neoplasm (MPN)-like phenotype that occasionally progressed to AML beginning ∼5 months post-transplant. The AML phenotype persisted upon serial transplantation (Gerrits et al., 2012). In a separate study, however, engraftment of HSCs ectopically expressing *miR-125a* declined over time in secondary recipients (Gerrits et al., 2012), suggesting that *miR-125a* cannot maintain long-term HSC self-renewal.

The pro-apoptotic gene, *Bak1*, was shown to be a *bona fide* target of *miR-125a* since its co-expression with *miR-125*a in 5-FU-treated BM blocked hematopoietic expansion *in vitro*. However, *Bak1-null* mice displayed a different hematopoietic phenotype, suggesting that *miR-125a* targets multiple genes to produce the myeloproliferative phenotype (Lindsten et al., 2000, 2003). In another study, *Lin28a*, a known target of *miR-125*, was suggested to partially mediate *miR-125*’s effects on lineage commitment since knocking down *Lin28a* in bone marrow cells increased myeloid cell number and reduced the number of B cells in mice, a phenotype reminiscent of the effects of *miR-125b* overexpression. However, leukemia did not develop in mice transplanted with *Lin28a* knockdown HSCs, suggesting that *Lin28a* is necessary, but not sufficient, for *miR-125*-driven leukemogenesis (Chaudhuri et al., 2012).

The *miR-125* overexpression studies have revealed numerous phenotypes including lineage bias, enhanced HSC function, and the induction of leukemia, raising questions regarding the physiologic role of *miR-125*. It appears that the varying phenotypes are likely due to differences in *miR-125* expression levels. Mice transplanted with human fetal liver (FL) cells expressing *miR-125b* at ∼1500-fold higher than endogenous levels develop a MPN-like disorder, while slightly lower levels of *miR-125b* expression (500-1000 fold) induce B- or T-cell acute lymphoblastic leukemias (B-ALL, T-ALL; Bousquet et al., 2010). Similarly, mice transplanted with 5-FU-treated BM cells expressing approximately 100-fold higher levels of *miR-125b-1* and *miR-125b-2* display expansion of all leukocyte lineages including lymphocytes, while mice expressing *miR-125b* at significantly higher levels (500-1000 fold) develop a MPN-like disorder. Although other experimental factors, such as starting HSPC populations and methods of handling of HSCs *in vitro* during viral transduction were not completely identical in these studies, the data suggest a strong gene dose:phenotype correlation.

Consistent with *miR-125b*’s leukemogenic function, co-transducing 5-FU-treated BM cells with *miR-125b* (overexpressed 500-fold) and the *BCR-ABL* fusion gene or mutant *C/EBP*α accelerated the development of leukemia (B-ALL, MPN, or mixed leukemia with *BCR-ABL,* and myeloid leukemia with *C/EBP*α; Bousquet et al., 2010; Enomoto et al., 2012). Furthermore, a number of studies have implicated *miR-125b* in the development of acute megakaryocytic leukemia (AMKL), particularly in patients with Down’s syndrome (DS). DS is characterized by trisomy 21; *miR-125b-2* is located on chromosome 21, and is expressed at markedly higher levels in patients with AMKL (both DS and non-DS related). Consistent with its role in determining the differentiation characteristics of AMKL, ectopic expression of *miR-125b* in K562 cells promotes megakaryocytic differentiation, and enforced expression of *miR-125b* in megakaryocytic progenitors (MPs) and megakaryocyte-erythroid progenitors (MEPs) isolated from mouse E12.5 FL leads to increases in the size, frequency, and re-plating capacity of megakaryocyte colony-forming units (Klusmann et al., 2010).

To better understand the mechanism by which *miR-125* promotes megakaryocytic differentiation, investigators have studied two *miR-125b* target genes, *DICER1* and the tumor suppressor *ST18*, both characterized as negative regulators of megakaryopoiesis. Knocking down *DICER1* or *ST18* in FL cells leads to megakaryocytic expansion, reminiscent of the phenotype observed upon *miR-125b* overexpression. However, the expansion was milder when compared to the *miR-125b*-driven phenotype, suggesting that other *miR-125b* targets are likely involved in this process. These studies also suggest that *miR-125b* aberrantly induces self-renewal in fetal committed MPs and MEPs, as *miR-125b-2* overexpression in these cells causes them to continue to proliferate in the presence of limited growth factors (e.g., TPO alone) in liquid culture for >1 month. Similar results were obtained using CD34^+^ cells from human cord blood (Klusmann et al., 2010), but not adult bone marrow MEPs, suggesting that *miR-125* plays distinct roles in adult and fetal hematopoiesis. These results provide a potential molecular basis for the robust association between DS and the development of AMKL, and likely explains the higher incidence of AMKL in pediatric populations relative to adults (Hasle, 2001).

In summary, the *miR-125* family regulates self-renewal, both in HSCs as well as fetal MP and MEPs. The variance in hematopoietic phenotypes induced by *miR-125* overexpression can be attributed, in large part, to the level of overexpression, with lower levels of *miR-125* expression regulating hematopoietic differentiation and proliferation and leading to myeloid (and sometimes T-cell) expansion at the expense of B cells, while the highest levels induce the development of a MPN-like phenotype that progresses to AML (**Table 1**). The relevance of these studies to human disease is worth considering, as *miR-125* is upregulated by no more than 90-fold in myeloid malignancies (Bousquet et al., 2008). Therefore, the high levels of *miR-125* expression in these studies raise concerns for possible non-physiologic and/or off-target effects that may not entirely reflect *miR-125*’s normal biological contribution to these processes. These concerns could be addressed by developing *miR-125* seed sequence mutants or through overexpression at levels similar to that observed in patient samples. In addition, complementary studies using additional knockdown approaches [e.g., locked nucleic acids (LNA’s) and sponges], and *miR-125*-targeted deletions could be used to determine whether *miR-125* is required for the development of leukemia in mouse models and if it regulates the function of leukemic stem cells or HSCs (Elmen et al., 2005; Ebert and Sharp, 2010; Chu et al., 2012). Unfortunately, the presence of multiple *miR-125* family members and their potentially overlapping and/or redundant functions make the generation of a *miR-125-null* mouse technically challenging. Finally, while the two *miR-125* paralogs share the same seed sequence, they differ in their mature sequence; thus, understanding the target genes mediating the specific effects of *miR-125a* and *miR-125b* will be an important area of investigation in the future.

**Table 1:** Summary of studies on *miR-125* in hematopoiesis and leukemia.

miRNA	Study	Cell type	1° transplant		2° transplant		Potential targets


			Timepoint analyzed	Hematopoietic phenotype	Timepoint analyzed	Hematopoietic phenotype	
*miR-125*b	Ooi et al. (2010), PNAS	HSC	2.5 months	35X expression: • Increased hematopoietic stem cell (HSC) number • Reduced HSC apoptosis • GM expansion • Reduced B cells	2.5 months	• GM expansion • Increased B cells • Lymphoproliferative disease with low penetrance	Pro-apoptotic *Klf13 and Bmf*
*miR-125b-1, miR-125b-2*	O’Connell et al. (2010), PNAS	5-FU-treated BM cells	2 months	100X expression: • Expansion of all WBCs including myeloid cells and lymphocytes 500–1000X expression: • Myeloproliferative disease (MPD), as evidenced by granulocyte/monocyte (GM) expansion • Reduced B cells, • Reduced platelets and RBCs, • Splenomegaly • Myeloid cell infiltration in the liver		• Not investigated	
*miR-125b*	Bousquet et al. (2008, 2010), JEM, PNAS	Fetal liver (FL) cells	4 months	500X expression: • T-ALL 1000X expression: • B-ALL 1500X expression: • Myeloproliferative neoplasm (MPN)		• Not investigated	*ABTB1, CBFB*
*miR-125a*	Guo et al. (2010), PNAS	5 × 10^5^ Total BM	4 months	1000X expression: • Enhanced reconstitution • >8-fold HSC expansion using limiting dilution analysis • The number of myeloid cells predominated with a reduced proportion of lymphoid cells	5 months	• Enhanced reconstitution	Pro-apoptotic *Bak1*
*miR-125a*	Gerrits et al. (2012), Blood	5–10 × 10^6^ 5-FU-treated BM cells	2.5–5 months	1500X expression: • Increased stem cell number • The number of GM and T-cells predominated with a reduced proportion of B lymphoid cells	1.5–6 months	• Unlike Guo et al. (2010), reduced engraftment of HSCs	

## miR-146

*miR-146a* is located on chromosomes 5 and 11 in the human and mouse genomes, respectively. Mature *miR-146a* is differentially expressed during hematopoietic development, with relatively low expression levels in HSPCs, and higher levels upon differentiation, especially in activated macrophages and T-cells (Boldin et al., 2011; Starczynowski et al., 2011; Yang et al., 2012), pointing to a potential role in hematopoiesis. However, *miR-146a* is expressed ∼1.5-fold higher in HSCs and in myeloid progenitors compared to other progenitor subtypes (Zhao and Starczynowski, 2014). *miR-146a* expression is regulated by several lineage-dependent transcription factors including the myeloid-specific transcription factor, PU.1 (Jurkin et al., 2010; Ghani et al., 2011), and the megakaryocyte-specific transcription factor, PLZF (Labbaye et al., 2008). In this section, we will focus on the role of *miR-146a* in the innate immune system, myelopoiesis and the development of myelodysplastic syndromes (MDS) and AML.

## *miR-146* AND INNATE IMMUNITY

**FIGURE 1:**
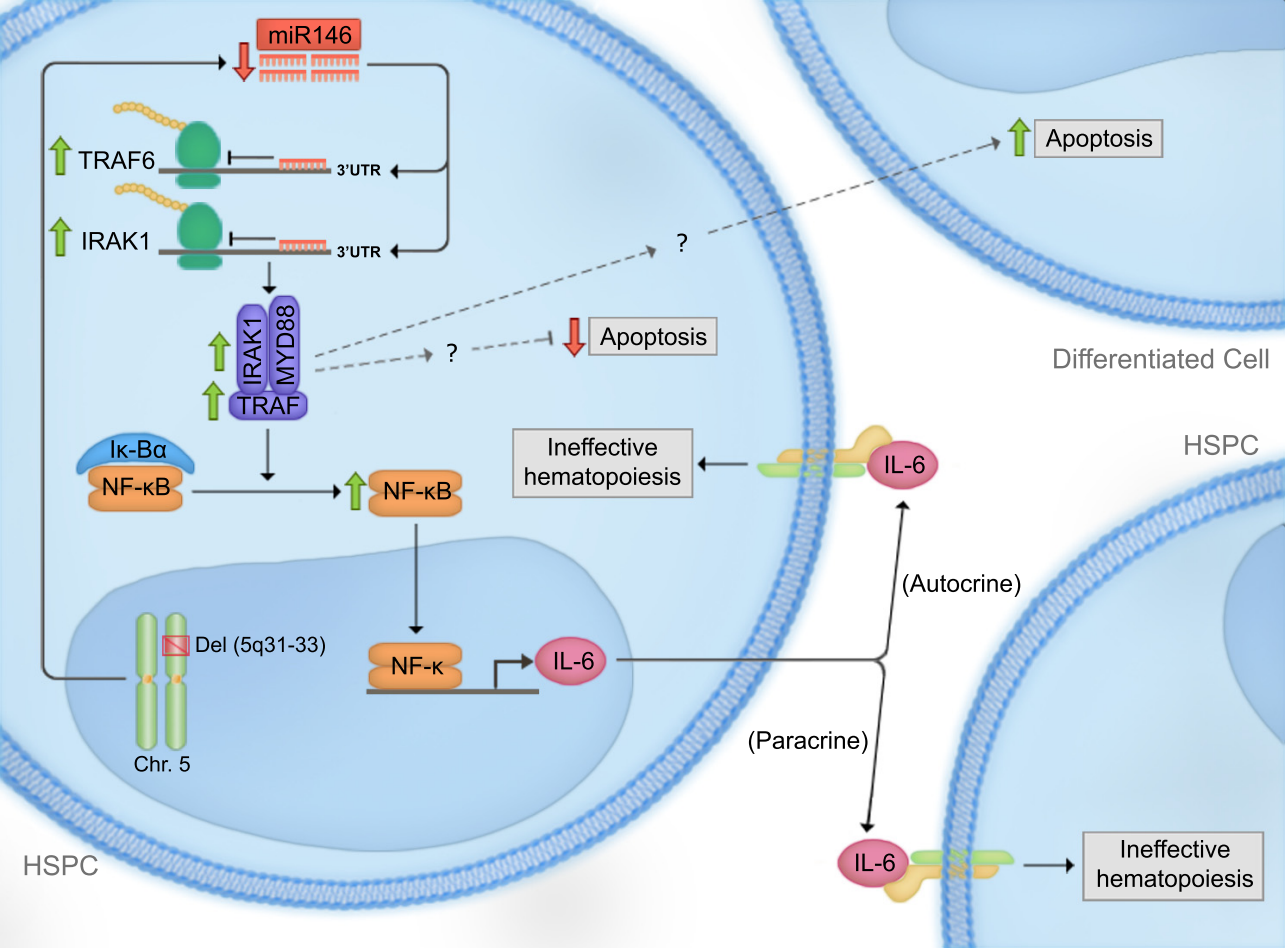
**Proposed model for the effect of *miR-146a* deficiency in the pathogenesis of del(5q) myelodysplastic syndromes (MDS).** Loss of one copy of *miR-146a* results in a decrease in its inhibitory effect on tumor necrosis factor receptor-associated factor 6 (*TRAF6*). *TRAF6-*mediated increase in IL-6 expression leads to autocrine and paracrine effects on HSPCs. Moreover, while *TRAF6* exerts autocrine anti-apoptotic roles in HSPCs, it leads to increased apoptosis in a paracrine manner. Thus, these observations could provide a possible mechanism for the observed bone marrow hypercellularity in MDS bone marrows, despite the presence of increased overall apoptosis. Downstream mediators of *TRAF6* leading to these effects are currently under investigation.

*miR-146a* has been shown to both regulate, and be regulated by, the NF-kB pathway, a critical mediator of inflammatory signaling, cell survival, differentiation, and proliferation (Silverman and Maniatis, 2001; Taganov et al., 2006; Hayden and Ghosh, 2008; **Figure 1**). NF-kB positively regulates *miR-146a* expression by binding two consensus-binding sites in the *miR-146a* promoter (Taganov et al., 2006). In contrast, *miR-146a* negatively regulates the NF-kB pathway by targeting of two positive regulators of NF-kB, tumor necrosis factor receptor-associated factor 6 (*TRAF6*) and IL-1 receptor-associated kinase-1 (*IRAK1*; Taganov et al., 2007; **Figure 1**). Consistent with *miR-146a*’s negative regulation of NF-kB signaling, *miR-146a* knockout mice display hypersensitivity in response to lipopolysaccharide (LPS) challenge, as evidenced by significantly elevated pro-inflammatory cytokine production (Boldin et al., 2011). Thus, *miR-146a* is an important negative regulator of innate immune activation.

## *miR-146a* AND NORMAL HEMATOPOIESIS

In normal hematopoiesis, *miR-146a* is a negative regulator of megakaryopoiesis and granulocyte/macrophage differentiation. Labbaye et al. (2008) showed that *miR-146a* is downregulated during megakaryopoiesis and that this downregulation is important for normal megakaryopoiesis. Overexpressing *miR-146a* in human CD34+ cells impaired megakaryocytic proliferation, differentiation, and maturation *in vitro*, while knocking down *miR-146* reversed this phenotype. These phenotypes appear to be mediated by at least two mechanisms: (i) circumventing the negative regulation of *miR-146a* by PLZF, a megakaryocytic lineage promoting transcriptional repressor, that binds to the *miR-146a* promoter (Labbaye et al., 2002), and (ii) directly inhibiting *CXCR4*, which is indispensible for megakaryopoiesis and contains a 3′UTR *miR-146a* binding site (Avecilla et al., 2004). Together, these studies suggest that megakaryopoiesis is controlled by the PLZF suppression of *miR-146a*, which in turn relieves the inhibition of *CXCR4* by *miR-146a*. In support of this model, Starczynowski et al. (2010) showed that knocking down *miR-146* using a miRNA decoy system in mouse HSPCs increased megakaryopoiesis *in vivo* through a TRAF6-dependent pathway, and that overexpressing TRAF6 in mouse bone marrow cells resulted in a similar phenotype (**Figure 1**).

Despite these data supporting a negative role for *miR-146a* in megakaryopoiesis, other studies have generated contradictory results. For instance, Opalinska et al. (2010) showed that overexpressing *miR-146a* in mouse HSCs fails to alter megakaryocyte development or platelet production *in vivo* and *in vitro*; these findings were confirmed in a separate study by Starczynowski et al. (2011). These conflicting results may stem from the attenuation of miRNA inhibition in the presence of abundant target transcripts, which has been previously described in other contexts (Arvey et al., 2010). As such, *miR-146a* expression may be sufficiently high in HSCs for maximal target gene inhibition; thus ectopic expression of *miR-146a* would not be expected to induce a substantial phenotypic change, while knocking down *miR-146a* would still be expected to release its inhibition of *TRAF6* and increase megakaryopoiesis, consistent with early findings of Starczynowski et al. (2010). Alternatively, the effect of *miR-146a* may be context-specific, since *miR-146a* overexpression in human HSPCs, specifically, has been shown to impair megakaryocytic differentiation (Labbaye et al., 2008). Bioinformatic approaches assigning *miR-146a* “inhibition indices” to its key target gene(s) (e.g., *TRAF6*) in human and mouse HSPCs might help resolve these paradoxical results (Arvey et al., 2010).

While *miR-146a* seems to a play a role in normal megakaryopoiesis, it also appears to be a negative regulator of granulocyte/macrophage differentiation in the context of aging. *miR-146a-null* mice display no detectable hematopoietic phenotypes under normal conditions during the first 2 months of life. While the absence of such a phenotype might be explained, in part, by compensatory effects of other members of the *miR-146* family, it might also be explained by a model in which *miR-146a* exerts its effects under non-homeostatic and/or stress conditions. Consistent with the latter, *miR-146a-null* mice produce increased numbers of granulocyte/monocyte (GM) cells with advancing age (Boldin et al., 2011). Such GM expansions have also been detected in young *miR-146a-null* mice upon repeated exposure to LPS (Zhao et al., 2013), suggesting that chronic inflammation may contribute to the myeloproliferative phenotype observed in aging *miR-146-null* mice. Furthermore, overexpressing *miR-146a* in young HSPCs results in opposite effects on granulopoiesis in young mice, suggesting that *miR-146a* functions as a transient positive regulator of myelopoiesis in young mice (Opalinska et al., 2010; Starczynowski et al., 2011). Consistent with this, transplantation of *miR-146a* knockdown HSPCs into lethally irradiated mice results in mild neutropenia (Starczynowski et al., 2010).

In addition to its regulation of myelopoiesis, *miR-146a* plays a role in HSC maintenance. *miR-146a-null* mice harbor decreased numbers of CD150+CD48- LT-HSCs by 8 months of age and HSC exhaustion by 12 months. In addition, a functional decline in HSCs is apparent as early as 2 months of age in competitive repopulation assays. These effects are predominantly cell-intrinsic since transplanting WT HSCs into *miR-146a-null* and normal donors results in minor differences. Intriguingly, *miR-146a*-deficient lymphocytes display a hyper-activated phenotype with dysregulated cytokine production, suggesting a lymphocyte-mediated mechanism for the reduction of HSC numbers. To test this possibility, *miR-146a* and *Rag1*, a gene required for lymphocyte maturation, were simultaneously deleted, leading to a partial rescue of the HSC exhaustion and the myeloproliferative phenotypes observed in *miR-146a-null* mice (Zhao et al., 2013). These findings are consistent with the systemic autoimmunity observed in some MDS patients (Nimer, 2008).

## *miR-146a* AND MYELOID MALIGNANCIES

*miR-146a* is located within the common deleted region (CDR) of del(5q) MDS. This deletion is associated with significantly reduced levels of *miR-146a* in human bone marrow cells compared to other subtypes of MDS. Similar reductions in expression are also observed with *miR-145*, which is also present in the 5q CDR (Starczynowski et al., 2010). To elucidate the role of these miRNAs in MDS, Starczynowski et al. (2010) knocked down *miR-146a* and *miR-145* in mouse HSPCs and transplanted them into lethally irradiated mice. Eight weeks following transplantation, recipient mice exhibited thrombocytosis, variable neutropenia, and hypolobated megakaryocytes in the bone marrow—all features observed in human del(5q) MDS patients. Using luciferase reporter assays, *TIRAP* and *TRAF6* were identified as target genes of *miR-145* and *miR-146a*, respectively. In order to determine whether *TRAF6* is a mediator of the del(5q) MDS phenotype, *TRAF6* was overexpressed in mouse bone marrow cells and transplanted into recipient mice. By 12 weeks, the recipient mice developed neutropenia, thrombocytosis, and increased hypolobated megakaryocytes in the bone marrow, and most progressed to bone marrow failure or AML at ≥5 months post-transplantation. In addition, knocking down *miR-146a* in *TRAF6-null* cells failed to increase megakaryocyte colony formation *in vitro*. Similarities between the *TRAF6*-induced mouse hematopoietic phenotype and human del(5q) MDS strongly suggest that down-regulation of *miR-146a* in HSPCs plays a critical role in the development of MDS, largely by inhibiting *TRAF6*. As *TRAF* proteins are key intermediaries in the activation of canonical NF-kB signaling pathway (Bradley and Pober, 2001), these data suggest that NF-kB may be downstream of *miR-146a* and responsible for mediating a significant part of the *miR-146a* phenotype. Support for this model was provided by experiments in which the NF-kB p50 subunit was knocked down and confirmed to rescue some aspects of the myeloproliferative phenotype of *miR-146a-null* mice (Zhao et al., 2011). Nevertheless, other signaling pathways, including non-canonical NF-kB pathways, contribute to the *miR-146a* phenotype as well (Etzrodt et al., 2012). For instance, *TRAF6* is known to regulate additional signaling pathways through its E3 ubiquitin ligase domain (Yang et al., 2009). Future studies are needed to identify pathways regulated by this activity of *TRAF6* in *miR-146a*-deficient HSPCs.

The co-occurrence of peripheral blood cytopenias with bone marrow hypercellularity and apoptosis is frequently observed in MDS (Boldin et al., 2011; Zhao et al., 2011). To explain this apparent paradox, it has been proposed that the increase in apoptosis is counterbalanced by a simultaneous increase in HSPC proliferation (Lepelley et al., 1996). This possibility was investigated in *TRAF6*-mutant mice that had progressed to bone marrow failure or AML. Non-*TRAF6* transduced regions of the bone marrow exhibited elevated levels of apoptosis relative to transduced regions. This finding raises the possibility that *TRAF6* protects HSPCs from cell death in a cell autonomous manner while simultaneously promoting apoptosis in non-*TRAF6*-expressing cells in a non-autonomous manner (**Figure 1**). Furthermore, megakaryocyte expansions observed in both the transduced and non-transduced cells in mice transplanted with *miR-146a* knockdown cells suggest a potential paracrine mechanism inducing thrombocytosis (**Figure 1**). To investigate this possibility, cytokines and growth factors involved in megakaryopoiesis were measured. Increased circulating IL-6, but not other cytokines, was detected in the serum of *miR-146a*-chimeric mice (Kishimoto, 2005). Concurrent transduction of dominant-negative *TRAF6* into mouse HSPCs reversed the phenotype, indicating that IL-6 induction by *miR-146a* knockdown is mediated through *TRAF6*. Interestingly, the leukemogenic activity of *TRAF6* was not affected when overexpressed in IL-6*-null* HSPCs, suggesting that a non-IL-6 dependent mechanism mediates the role of *TRAF6* in the development of leukemia.

Additionally, the role of *miR-146a* as a tumor suppressor in MDS is supported by the development of pancytopenia and myeloproliferation involving the spleen, bone marrow, and secondary lymphoid organs in aging *miR-146-null* mice. Consistent with its negative regulation of myeloproliferation, *miR-146a-null* BM derived macrophages (BMDM) exhibit increased rates of proliferation, likely due to elevated levels of M-CSF receptor (*CSF1R*) expression and M-CSF signaling (Boldin et al., 2011).

In summary, *miR-146a* regulates HSC maintenance as well as megakaryocytic differentiation. It also regulates GM differentiation in an age-dependent manner. Moreover, *miR-146a* down-regulation contributes to the development of del(5q) MDS and promotes disease progression to AML through the *TRAF6*-mediated induction of NF-kB and apoptosis. Additional studies are necessary to elucidate the relative contribution of *miR-146a* to del(5q) MDS pathogenesis by investigating the potential additive or synergistic effects of *miR-146a* with other genes located in the 5q deleted region (e.g., ribosomal protein RPS14; Ebert et al., 2008) using both mouse models and primary MDS patient samples.

## miR-155

Human *miR-155*, located on human chromosome 21, resides in a spliced and polyadenylated non-coding RNA transcript called the B-cell integration cluster (BIC; Tam, 2001). BIC is an evolutionarily conserved RNA that was initially shown to cooperate with c-Myc to induce lymphomas in chickens when aberrantly activated by pro-viral integrations at a common retroviral integration site (Tam et al., 1997; Tam and Dahlberg, 2006). More recently, studies suggest that *miR-155* may also play a role in mediating leukemogenesis. While the mechanisms underlying the transformative activity of this non-coding RNA were a mystery for some time, it has now become clear that BIC contains the pre-*miR-155* sequence and that it mediates its oncogenic functions by giving rise to the mature *miR-155* transcript (Lagos-Quintana et al., 2002; van den Berg et al., 2003; Eis et al., 2005; Kluiver et al., 2005). In this section, we will review studies that have established a role for *miR-155* in the pathogenesis of myeloid malignancies.

## *miR-155* IN NORMAL AND MALIGNANT MYELOPOIESIS

During steady state hematopoiesis, *miR-155* is expressed at high levels in HSPCs and at low levels in most mature hematopoietic cells; for example, *miR-155* is expressed at high levels in erythroblasts relative to maturing erythroid progenitors (Georgantas et al., 2007; Masaki et al., 2007; O’Connell et al., 2008). Moreover, *miR-155* expression increases during B- (Tam, 2001; van den Berg et al., 2003; Eis et al., 2005; Tam and Dahlberg, 2006; Masaki et al., 2007; Garzon et al., 2008b) and T-cell activation (Rodriguez et al., 2007), and upon exposure of innate immune cells (e.g. monocytes) to inflammatory stimuli such as LPS (Gorgoni et al., 2002; Hayden and Ghosh, 2008; O’Connell et al., 2009). The latter likely explains *miR-155’*s role in mediating innate immune responses (Georgantas et al., 2007).

Overexpressing *miR-155* in HSCs leads to the expansion of GM cells, extramedullary hematopoiesis, and the development of GM cells with morphologic dysplasia in C57BL/6 mice, compatible with a myeloproliferative-like disorder (Garzon et al., 2008a,b; Jongen-Lavrencic et al., 2008). *miR-155* is also expressed at low levels in mature erythroid cells, and overexpression of miR-155 in HSCs is associated with a reduction in Ter119+ erythroid progenitors in the mouse bone marrow. This latter finding is consistent with observations in human CD34+ cells overexpressing *miR-155* (Georgantas et al., 2007). *miR-155* may concurrently inhibit megakaryopoiesis, as *miRNA-155*-transduced K562 cells treated with hemin, an inducer of megakaryocytic differentiation, exhibit reduced expression of CD41 (Georgantas et al., 2007). While these studies collectively indicate that *miR-155* regulates myeloid lineage commitment, the mechanisms by which *miR-155* exerts its effects – whether by negatively regulating apoptosis, promoting commitment to the common myeloid progenitor (CMP) lineage in HSPCs, or by increasing the rate of proliferation among myeloid progenitors or their maturing progeny – remains unresolved and will need to be explored in future studies.

Consistent with ectopic overexpression of *miR-155* inducing a myeloproliferative phenotype, several studies have shown the upregulation of *miR-155* in the bone marrow of NPM1 and FLT3-ITD-mutant AML patients (Garzon et al., 2008a,b; Jongen-Lavrencic et al., 2008). It is also possible that the *miR-155* myeloproliferative phenotype was observed due to the effects of miR-155 overexpression being only assessed in the transplantation setting, which requires lethal irradiation, the induction of strong inflammatory responses, and the up-regulation of *miR-155* expression in the bone marrow of recipient mice. Consistent with this idea, altered myeloid phenotypes have not been observed in the bone marrow or peripheral blood of *miR-155-null* mice (Rodriguez et al., 2007).

## MECHANISM OF ACTION OF *miR-155*

To determine which *miR-155* target genes are required to induce the *miR-155* overexpression phenotype, O’Connell et al. (2008) overexpressed *miR-155* in RAW 264.7 myeloid cells and showed reductions in the transcripts of several genes (*Bach1*, *Sla, Cutl1*, *Csf1r*, *Jarid2*, *Cebp*β, *PU.1*, *Arntl*, *Hif1*α, and *Picalm*) known to play critical roles in hematopoiesis; subsequent studies have established that *miR-155* directly inhibits src homology 2 domain-containing inositol-5-phosphatase (*SHIP1*) as well as CCAAT-enhancer-binding protein-beta (*CEBP-*β) to mediate leukemogenesis (Gorgoni et al., 2002; O’Connell et al., 2009). The functional link between *SHIP1* and *miR-155* was strongly suggested by showing that knocking down *SHIP1* or overexpressing *miR-155* in HSPCs induces similar myeloproliferative phenotypes characterized by increased numbers of CD11b+ myeloid cells in the bone marrow and spleen, decreased erythropoiesis in the bone marrow, and splenomegaly (O’Connell et al., 2009). In contrast to *miR-155*, which may require previous irradiation to mediate myeloproliferation, *SHIP1*-null mice display a myeloproliferative phenotype in the absence of stress/inflammatory stimuli. This is likely due to higher baseline levels of cell-intrinsic inflammation in *SHIP1-null* mice as they are hyper-responsive to cytokine stimulation *in vitro* (Helgason et al., 2000) due to the loss of SHIP1’s negative regulation of cytokine signaling (Kalesnikoff et al., 2003; Leung et al., 2009). This functional interaction is of great interest since *SHIP1* was previously shown to be a tumor suppressor in AML (Luo et al., 2003).

**FIGURE 2:**
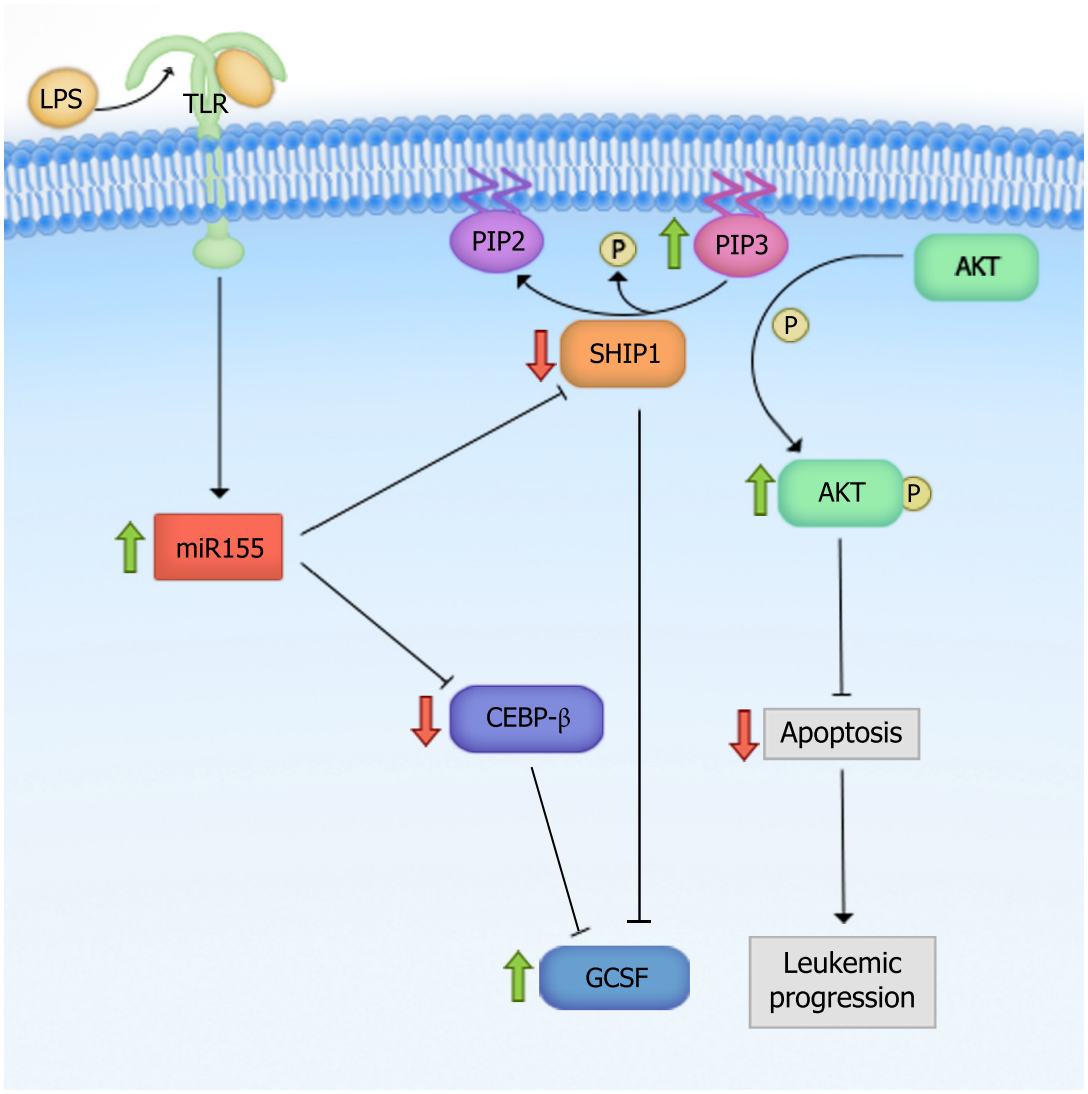
**Proposed mechanism for *miR-155*-mediated myeloid leukemogenesis.** Overexpression of *miR-155* leads to the activation of the PI3K-Akt pathway through negative regulation of Src Homology 2 domain-containing Inositol-5-Phosphatase (*SHIP1*). *SHIP1* is a phosphatase that mediates the conversion of phosphatidylinositol triphosphate (PIP3) to phosphatidylinositol diphosphate (PIP2). PIP3 normally acts as a docking site for signaling molecules in the PI3K-Akt pathway and helps relay the signal. Upon *miR-155* overexpression and thus, SHIP1 downregulation, PIP3 level is increased, which leads to the activation of the PI3K-Akt pathway. Green arrows indicate increased, and red arrows decreased activity upon *miR-155* overexpression.

*SHIP1* is a phosphatase that mediates the conversion of phosphatidylinositol triphosphate (PIP3) to phosphatidylinositol diphosphate (PIP2). PIP3 normally acts as a docking site for signaling molecules in the PI3K-Akt pathway (e.g., Akt and PLC) and helps relay the signal (**Figure 2**). Thus, *SHIP1* blocks the activation of the PI3K-Akt pathway (Damen et al., 1996; Ono et al., 1996). *SHIP1*’s ability to suppress the development of AML is probably mediated through this pathway (Luo et al., 2003). Since SHIP1 has been shown to negatively regulate PI3K/Akt signaling, it would be interesting to investigate whether *miR-155*-induced phenotypes depend on the activation of this pathway. Moreover, in these studies, the phenotypes of *SHIP1* and *miR-155* were investigated independently. However, the importance of *SHIP1* in the *miR-155* overexpression phenotype has not been explored by testing the ability of *miR-155* to induce phenotypes in the context of *SHIP1* loss-of-function or deficiency. It is also unclear at which stage of hematopoietic development the *miR-155*:*SHIP1* interaction is required to induce these phenotypes. It would presumably be in an early progenitor population based on *miR-155*’s high levels of expression in HSPCs.

Another *miR-155* target gene, *CEBP-*β, appears to ensure that the hyperproliferation under stress is myeloid in nature. *CEBP-*β is a transcription factor involved in macrophage activation and the induction of pro-inflammatory cytokines and acute phase reactants (Gorgoni et al., 2002). In one study, LNA-induced *in vivo* silencing of *miR-155* in the splenocytes of LPS-treated mice led to the de-repression of *CEBP-*β compared to LNA-control LPS-treated mice. Moreover, antagonism of *miR-155* in an AML cell line was also accompanied by a reduction in the inflammatory cytokine G-CSF (Worm et al., 2009). These findings suggest that *miR-155* overexpression induces GM expansion by targeting *CEBP-*β. However, it is unclear whether such a reduction in G-CSF production is dependent on *CEBP-*β, *SHIP1*, or both. We speculate that it is likely mediated through *SHIP1* since *SHIP1*-deficient mice have been shown to exhibit increased G-CSF production (**Figure 2**; Hazen et al., 2009).

Interestingly, *miR-155*-mediated hematopoietic malignancies exhibit longer latencies compared to more aggressive miRNA leukemia models, such as those induced by *miR-125* overexpression. This raises the possibility that additional mutations are required for full transformation. Intriguingly, *miR-155* targets mismatch repair genes such as *hMLH1*, *hMSH2*, and *hMSH6* (Valeri et al., 2010), as well as cell-cycle regulators such as *WEE1* (Tili et al., 2011). Thus, *miR-155* may increase spontaneous mutation rates in HSPCs, in agreement with observations made in some solid tumor cell lines (Mantovani et al., 2008; Valeri et al., 2010; Tili et al., 2011). It would be interesting to investigate this hypothesis in the context of AML and to determine whehter such mutations are required for the full manifestation of disease phenotypes.

While the cumulative data indicate that *miR-155* can initiate early events in myeloid leukemogenesis, it is not clear whether *miR-155* is required for leukemic progression or maintenance. Knocking down *miR-155* in fully transformed AML cells or reversibly expressing *miR-155* in a progressive model of AML would help address these important questions. If *miR-155* can be shown to regulate disease maintenance or resistance to therapy, it would be an excellent target in the treatment of AML.

## miR-142

*miR-142* is located on human chromosome 17. While *miR-142* is expressed in all hematopoietic tissues including the bone marrow, spleen and thymus, it is not expressed in non-hematopoietic tissues. Perhaps it is not surprising that the first role ascribed to *miR-142* was in promoting the development of the T-cell and myeloid lineages (Chen et al., 2004). *miR-142*’s importance as a regulator of hematopoiesis was recently underscored by the fact that it is the only miRNA that is recurrently mutated in AML (Cancer Genome Atlas Research Network, 2013).

## *miR-142* IN NORMAL AND MALIGNANT HEMATOPOIESIS

*miR-142* regulates normal hematopoiesis as well as the development of lymphoid and myeloid leukemias. By enforcing the expression of *miR-142* in Lin^-^ mouse bone marrow cells, Chen et al. (2004) showed that *miR-142* increases the absolute numbers of T-cells, leads to a minimal decrease in B-cell differentiation (CD19^+^), and slightly reduces the number of Mac1^+^ Gr1^-^ myeloid cells. *miR-142* also appears to play a role in the development of mature lymphoid malignancies, as the *miR-142* locus is 50bp from the breakpoint of t(8;17), a cytogenetic alteration present in a subset of aggressive mature B-cell leukemias (Gauwerky et al., 1989). This suggests that *MYC*, located on chromosome 8, is translocated and regulated by the upstream *miR-142* promoter (Lagos-Quintana et al., 2002). Interestingly, *miR-142-3p* is significantly downregulated in ALL patients expressing the *MLL-AF4* fusion gene. Ectopic expression of *miR-142-3p* in *MLL-AF4^+^* cell lines supresses cell proliferation, induces apoptosis, and down-regulates multiple genes known to regulate self-renewal including *MLL-AF4*, *HOXA9*, *HOXA7*, and *HOXA10*. Thus, *miR-142-3p* likely functions as a tumor suppressor in *MLL-AF4^+^* ALL (Dou et al., 2013).

In addition to its role in lymphopoiesis and lymphoid malignancies, *miR-142* also regulates myeloid differentiation and the development of AML. It is up-regulated during myeloid differentiation in both normal and leukemic HSPCs. Overexpressing *miR-142* promotes phorbol-12-myristate-13-acetate (PMA)-induced monocytic and all-trans retinoic acid (ATRA)-induced granulocytic differentiation in AML cell lines by directly targeting the *cyclin T2* (*CCNT2*) and “TGFβ–activated kinase 1/*MAP3K7* binding protein 2” (*TAB2*) transcripts. In addition, *miR-142-3p* levels are significantly reduced in CD34+ cells from primary AML samples. This decrease is associated with the increased expression of *CCNT2* and *TAB2*, two predicted *miR-142* targets. Conversely, enforced expression of *miR-142-3p* in HSPCs from healthy controls and AML patients down-regulates the expression of *CCNT2* and *TAB2* and promotes myeloid differentiation (Wang et al., 2012b). Thus, reductions in *miR-142-3p* likely result in the differentiation blockade that is characteristic of AML. In support of this model, independent expression profiling studies revealed that *miR-142-3p* is down-regulated in peripheral blood mononuclear cells (PBMCs) from AML patients (Wang et al., 2012a). Furthermore, higher *miR-142* expression correlates with a better prognosis in patients with intermediate-risk AML (Wang et al., 2012b; Dahlhaus et al., 2013). Perhaps the most compelling evidence supporting the role of *miR-142-3p* in leukemogenesis comes from RNA-sequencing studies of AML. Of the samples examined, 7/200 (3.5%) harbored mutations in miRNAs, of which 4/7 (57%) were present in the seed sequence of *miR-142-3p*. Other non-recurrent mutations identified in miRNAs were found in *miR-516b1*, *miR-1267*, *miR-891a,* and *miR-632*. Additional studies will be required to elucidate how *miR-142* and other mutated miRNAs contribute to AML pathogenesis. This is particularly important because the novel seed sequences generated are predicted to alter target specificity and it is unclear whether these mutations are loss- and/or gain-of-function in nature (Cancer Genome Atlas Research Network, 2013).

## miR-29

*miR-29* family members have been shown to function both as tumor suppressors and oncogenes in myeloid malignancies. The *miR-29* family consists of three members, *miR-29a*, *miR-29b*, and *miR-29c*. The *miR-29a/miR-29b-1* and *miR-29b-2/miR-29c* clusters are present on chromosomes 7q32 and 1q23, respectively (Hwang et al., 2007; Garzon et al., 2009a). In this section, we describe the role of *miR-29* in regulating epigenetic modifiers, cellular proliferation, apoptosis, and hematopoietic differentiation, and the role of these functional changes in AML pathogenesis.

## *miR-29* AND EPIGENETIC REGULATION

Myeloid malignancies frequently exhibit epigenetic alterations, and subsets of patients with MDS, MPN, and AML have been shown to harbor activating mutations and loss-of-function mutations in master epigenetic regulators such as DNA methyltransferase 3 (*DNMT3*) and *TET*-eleven translocation 2 (*TET2*); the combination of these mutations account for 33% of the somatic mutations identified in AML (Delhommeau et al., 2009;Langemeijer et al., 2009; Tefferi et al., 2009a,b,c; Patel et al., 2012). Interestingly, the up-regulation of *DNMT* transcripts (including *DNMT1, 3A, and 3B*) and reductions in *TET2* enzymatic activity have been identified in patients harboring wild-type *DNMT* and *TET2*, suggesting that their activity is regulated by transcriptional and post-transcriptional regulatory mechanisms (Mizuno et al., 2001; Ko et al., 2010). In a subset of these patients, *IDH* mutations are likely to account for the dysregulation of *TET2* activity, while other unidentified factors also likely alter *TET2* activity (Shih et al., 2012).

**FIGURE 3:**
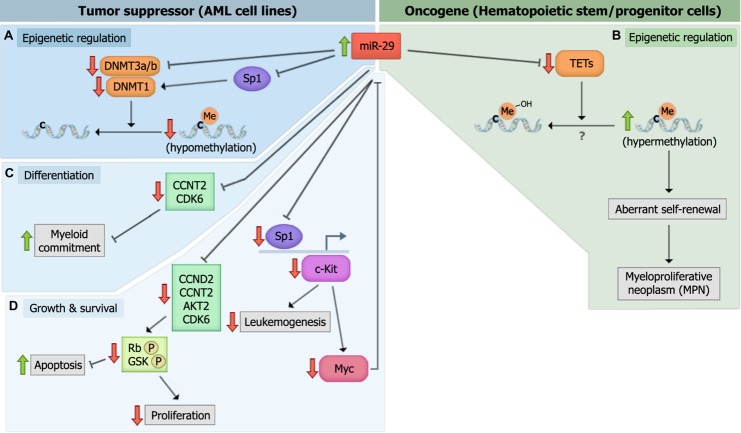
***miR-29* family members target multiple genes to mediate their biologic effects. (A)** *miR-29* directly targets DNA methyltransferase 3 (*DNMT3a)* and *DNMT3b*, and indirectly targets *DNMT1* by down-regulating Sp1, to mediate global DNA hypomethylation in acute myeloid leukemia (AML) cells. **(B)** *miR-29* targets *TET*-eleven translocation (*TET)* family members, *TET1*, *TET2*, and *TET*3, which may or may not mediate global DNA hypermethylation. The down-regulation of *TETs* induces aberrant self-renewal and the development of myeloproliferative neoplasm (MPN’s). **(C)** *miR-29* targets *cyclin T2* (*CCNT2*) and *CDK6* to promote myeloid differentiation. **(D)** *miR-29* promotes the down-regulation of *c-Kit* during normal hematopoiesis. However, the down-regulation of *miR-29* in malignant hematopoiesis promotes c-kit-driven AML by up-regulating Sp1, which increases c-Kit expression and suppresses *miR-29* expression.

The down-regulation of *miR-29b* is thought to promote DNA hypermethylation in AML since *miR-29b* can directly target *DNMT3A, DNMT3B*, and *Sp1* (a transcriptional regulator of *DNMT1*; **Figure 3A**; Garzon et al., 2009b). This link between miR-29 expression and methylation status in AML cells prompted the evaluation of *miR-29b* as a therapeutic target in AML. Investigators have shown that *miR-29b* oligonucleotide mimics recapitulate the effects of hypomethylating agents, 5-azacytidine and decitabine, by demethylating the promoters of tumor suppressors estrogen receptor 1 (*ESR1*) and *p15^INK4b^,* and by promoting the re-expression of these genes in AML cell lines (Garzon et al., 2009b). These results indicate that *miR-29b* oligonucleotides may be an effective therapeutic strategy in the treatment of AML.

In contrast to *DNMTs*, which methylate CpG islands and generally mediate transcriptional suppression, *TET* family members (*TET1, TET2, and TET3*) demethylate DNA by catalyzing the conversion of 5-methylcytosine (5mC) into intermediates of DNA methylation, including 5-hydroxymethylcytosine (5hmC), 5-formylcytosine (5fC), and/or 5-carboxylcytosine (5caC; He et al., 2011; Ito et al., 2011). Cheng et al. (2013) found that overexpressing any of the three *miR-29* family members in mouse bone marrow cells reduced the level of *TET2* as well as its metabolic by-product, 5hmC (**Figure 3B**). The reduction in 5hmC was rescued by ectopically expressing *TET2*. Of note, however, reductions in 5hmC levels in the wild-type mice transplanted with *miR-29b*-transduced bone marrow cells were attributed to global DNA hypermethylation, while direct evaluation of the global DNA methylation (GDM) status of these cells was not performed. Performing mass spectrometry on *miR-29* overexpressing cells and evaluating whether the reductions in 5hmC correlate with increases in GDM are critical, as studies identifying reduced 5hmC as a surrogate for global DNA hypomethylation have yielded conflicting results (Ko et al., 2010; Shih et al., 2012). It would also be interesting to evaluate the levels of the *DNMTs, TETs*, and GDM simultaneously upon *miR-29* overexpression in leukemic blasts and normal HSPCs. This would help elucidate whether the effect of *miR-29* on *DNMTs* and *TETs* are cell context-specific, or whether *DNMTs* and *TETs* function to promote the methylation and demethylation of distinct target genes simultaneously.

## *miR-29* AND MYELOID DIFFERENTIATION

Numerous studies have demonstrated that *miR-29* family members are regulators of myeloid differentiation. Han et al. (2010) showed that transplanting mice with HSPCs overexpressing *miR-29a* results in increased myeloid and reduced lymphoid chimerism 8–12 weeks post-transplant, and is accompanied by splenomegaly as well as megakaryocytic and granulocytic hyperplasia in the bone marrow and spleen, consistent with a myeloproliferative phenotype. Similarly, Wang et al. (2012b) reported that *miR-29a* expression increases with PMA-induced monocytic differentiation and ATRA-induced granulocytic differentiation in AML cell lines. The latter study showed that the increase in *miR-29a* expression was associated with reductions in *CDK6* levels upon myeloid differentiation and reductions in *CCNT2* upon monocytic differentiation. In this study, *CCNT2* and *CDK6* were shown to be authentic targets of *miR-29a*, and their reduced expression was necessary and sufficient for enhanced PMA and ATRA-induced myeloid differentiation. These findings were validated in human samples, as *miR-29a* overexpression partially reversed the differentiation arrest phenotype in primary AML blasts. Moreover, enforced expression of *miR-29a* in healthy CD34+ cells promoted monocytic and granulocytic differentiation (Wang et al., 2012b). These findings indicate that *miR-29a* regulates myeloid differentiation, at least partially through the targeting of *CCNT2* and *CDK6* (**Figure 3C**). Additional studies have shown that overexpressing another member of the *miR-29* family, *miR-29b*, promoted partial differentiation of an AML cell line, while both *miR-29b* and *miR-29c* promoted myeloid differentiation in AML patient CD34+ blasts (Fujimoto et al., 2007; Garzon et al., 2009b; Cheng et al., 2013; Gong et al., 2014). Furthermore, transplanting mouse bone marrow cells overexpressing *miR-29b* led to biased myeloid differentiation, splenomegaly, and an increased percentage of donor-derived monocytes in the bone marrow, inducing a chronic myelomonocytic leukemia (CMML)-like disease (Cheng et al., 2013). Given these findings, it would be interesting to determine whether *miR-29* oligonucleotides can act synergistically with ATRA, arsenic trioxide, or ATRA-arsenic trioxide combinations in primary AML patient blasts to evaluate the potential therapeutic efficacy of *miR-29* mimics in the treatment of acute promyelocytic leukemia (APL). Combinatorial regimens including *miR-29* with cytarabine and/or daunorubicin could also help elucidate its therapeutic efficacy as a stimulant of myeloid differentiation in non-APL myeloid leukemias.

## *miR-29* IN CELLULAR PROLIFERATION AND APOPTOSIS

In addition to their roles in epigenetic regulation and differentiation, *miR-29a* and *miR-29b* have been shown to regulate a number of cellular processes. A transcriptomal analysis of human leukemia cell lines transfected with synthetic *miR-29b* revealed the enrichment of genes that regulate apoptosis, cell cycle progression, and cellular proliferation. Altered expression of some of these genes (e.g., *MCL-1* and *CDK6)* was confirmed in primary AML blasts following transfection with *miR-29b* mimics (Garzon et al., 2009a). These changes were also accompanied by a reduction in phosphorylated Rb (pRb) due to the *miR-29* targeting of *CDK6* and *CCNT2*. Similar findings were generated by Gong et al. (2014), who showed that overexpressing *miR-29* family members in AML cell lines stimulates apoptosis and inhibits the G1 to S phase cell cycle transition, phenotypes attributed to the *miR-29* targeting of *CCND2* and *AKT2* (Gong et al., 2014; **Figure 3D**). In contrast, Han et al. (2010) found that overexpressing *miR-29a* in 293T cells enhanced cell cycle entry without altering apoptosis. In addition, mice overexpressing *miR-29a* demonstrated a developmental stage-specific increase in proliferation, as multipotent progenitors (MPPs) showed increased numbers of cycling cells while more committed GMP and CMPs did not (Han et al., 2010). These findings suggest that the cellular consequences of *miR-29* overexpression are cell-type specific and may depend on the state of differentiation and/or transformation.

## *miR-29* AS AN ONCOGENE AND TUMOR SUPPRESSOR

While overexpression studies in leukemic cell lines have shown that *miR-29* family members are able to regulate leukemic cell growth and survival, other studies have revealed the leukemogenic potential of the *miR-29* family. Han et al. (2010) showed that CMPs and GMPs from mice overexpressing *miR-29a* establish long-term, differentiating grafts in transplantation studies, suggesting that *miR-29a* overexpression is sufficient to induce aberrant self-renewal in CMPs and GMPs, but not a fully transformed phenotype. Mice serially transplanted with bulk splenocytes or bone marrow cells from these *miR-29a* overexpressing mice developed organomegaly and increased myeloid blasts in the bone marrow and spleen, consistent with transformation into AML. Similarly, Cheng et al. (2013) showed that mice transplanted with bone marrow cells overexpressing *miR-29b* developed splenomegaly and an increase in myeloid bias index, which are accompanied by reductions in 5hmC levels. Interestingly, the authors found that expression of *TET2* partially rescued this malignant phenotype. It remains unclear whether overexpression of *TET1*, *TET2*, and *TET*3 together could completely rescue the phenotype. All of the *TET* family members regulate DNA methylation and are *bona fide* targets of *miR-29b,* making this a possibility. Nevertheless, these studies indicate that the enforced expression of *miR-29a* in immature hematopoietic cells drives leukemic transformation, and that this phenotype can be attributed, at least in part, to the targeting of *TET2*. Moreover, the Han et al. (2010) study emphasizes the need to investigate the function of potential leukemia-modifying genes in a cell-specific manner since the functional consequences can vary dramatically based on cell context. This point is well-illustrated by the finding that *miR-29a* is up-regulated in patient LSC-enriched fractions and not in non-LSC fractions, which in contrast to other studies that have shown reduced *miR-29a* levels in whole PBMNC’s and BM CD34+ leukemic blasts (Wang et al., 2012b; Gong et al., 2014). Thus, the down-regulation of *miR-29’*s observed in previous studies may be due to the inclusion and dominance of non-LSC’s in the evaluated cells.

Consistent with *miR-29*’s role in promoting leukemogenesis, it appears that inhibiting *miR-29* in established blasts may be a promising therapeutic strategy. Injection of precursor *miR-29b* oligonucleotides in mice engrafted with K562 tumors significantly reduce their size (Garzon et al., 2009a). Subsequent studies showed that a transferrin-conjugated nanoparticle delivery system for synthetic *miR-29b* (Tf-NP-*miR-29b*) suppressed AML growth, impaired colony formation, and reduced cell viability in AML patient samples. Tf-NP-*miR-29b* also reduced spleen weight and increased overall survival in NSG mice transplanted with AML cell lines (Huang et al., 2013). Similarly, intravenous injections of NOD/SCID mice engrafted with AML with viral particles expressing *miR-29a*, *-29b*, and *-29c* reduced the number of CD33+ leukemic cells in the bone marrow and spleen by inhibiting proliferation and stimulating apoptosis (Gong et al., 2014). While the mechanism of leukemic blast clearance was not examined in these studies, others have suggested that the therapeutic effect of *miR-29b* may be mediated through its regulation of c-Kit through a feedback-loop (Liu et al., 2010). This is not surprising considering that increased c-Kit activation, either through stimulation by its ligand or secondary gain-of-function mutations, has been shown to drive leukemogenesis. In this study, c-Kit was shown to activate the transcription factor, MYC, which subsequently binds to, and inhibits, the *miR-29b* promoter (Liu et al., 2010). The reduction in *miR-29b* expression was shown to positively regulate Sp1 levels, thus allowing the formation of the Sp1/NF-kB complex, which binds regulatory sequences to increase c-Kit expression. It was also shown to positively regulate the formation of a complex with HDAC1, which further suppresses *miR-29b* expression. In support of this model, pharmacologic inhibition of c-Kit with bortezomib in NOD/SCID mice transplanted with *c-Kit* mutant AML cells (FDC-P1/*KIT^mu^*^t^) abrogated *c-Kit* mRNA levels and increased *miR-29b* expression. In addition, FDC-P1/*KIT^mut^* cells exhibited reduced tumor size, tumor weight, and engraftment efficiency upon transfection with synthetic *miR-29b*. These findings demonstrate the therapeutic potential of *miR-29* oligonucleotides and suggest that the down-regulation of *miR-29b* is particularly critical to leukemogenesis in c-Kit-driven AML (Liu et al., 2010).

## CONCLUSION AND FUTURE DIRECTIONS

**FIGURE 4:**
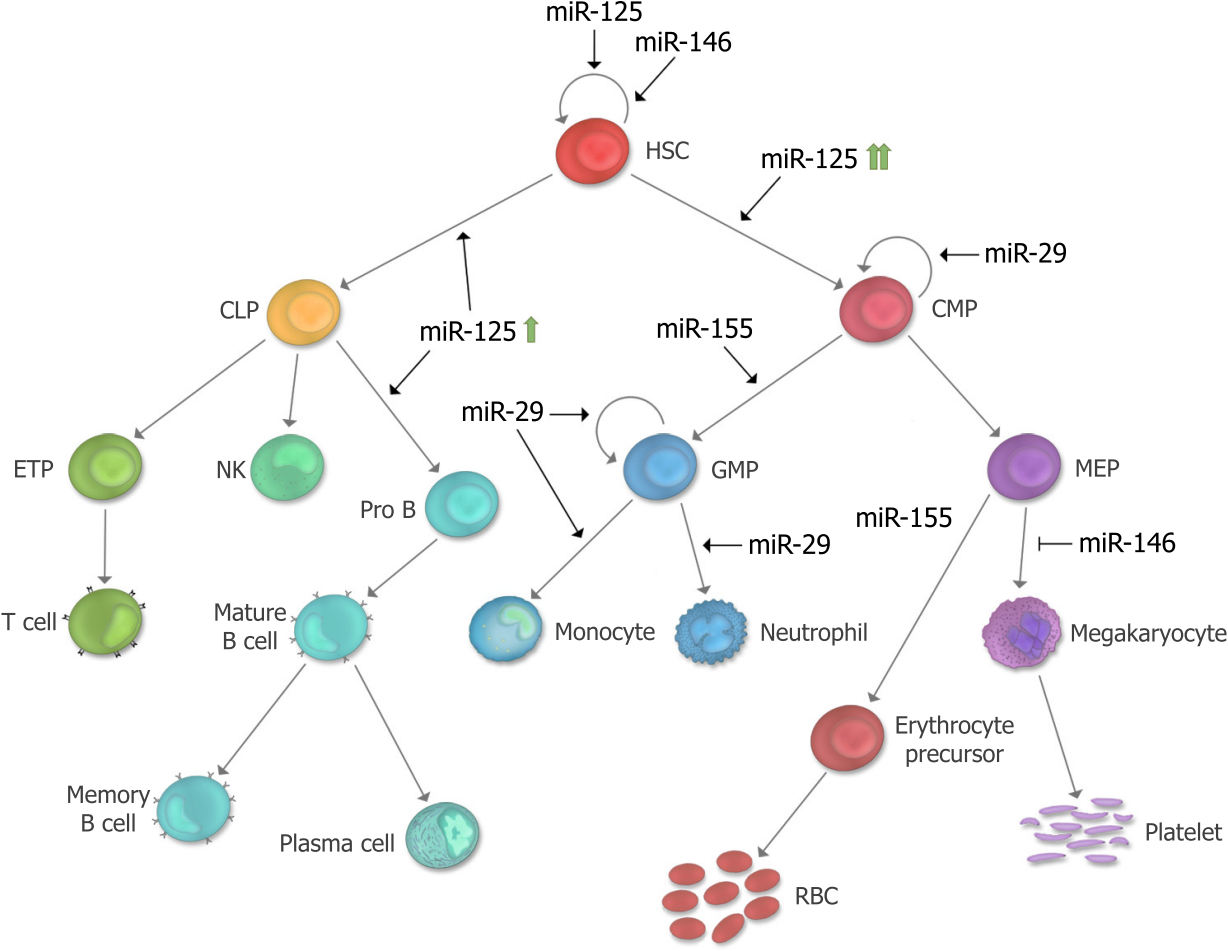
**Summary of proposed developmental stages at which microRNAs (miRNAs) functionally contribute in leukemogenesis.** The stages at which each of the five miRNAs described in this review exert their effect in hematopoiesis and leukemogensis are depicted. *miR-125* is a positive regulator of HSC self-renewal, and depending on its level of expression, can lead to biased myeloid or lymphoid differentiation. *miR-146a* regulates HSC maintenance as well as megakaryocytic differentiation. *miR-155* is a positive regulator of GM differentiation and a negative regulator of erythroid differentiation. *miR-29* is a positive regulator of myeloid differentiation, and overexpression can induce aberrant self-renewal of GMP’s. HSC, hematopoietic stem cell; CMP, common myeloid progenitor; MEP, megakaryocyte erythroid progenitor; RBC, red blood cell; GMP, granulocyte macrophage progenitor; CLP, common lymphoid progenitor; ETP, early thymic progenitor; NK, Natural Killer cell.

Although numerous miRNAs are dysregulated in AML, only a few have been shown to play functional roles in myeloid leukemogenesis (**Table 2**). Collectively, the data indicate that these miRNAs induce hematopoietic malignancies by exerting their biologic effects in developmental stage-specific manners. A summary of the predicted stage at which each of these miRNAs exerts its leukemogenic function is indicated in **Figure 4**. It is also worth mentioning that miRNAs may act in non-hematopoietic cell-intrinsic manners to promote leukemogenesis, as demonstrated by Raaijmakers et al. (2010), who showed that deleting the miRNA processing enzyme, *DICER1*, in mouse osteoprogenitors induces MDS which progresses to AML. These studies suggest that the absence of one or more miRNAs in bone marrow stromal cells may increase the predisposition towards developing myeloid malignancies (Raaijmakers et al., 2010).

**Table 2:** Summary of miRNAs with validated functional relevance in the pathogenesis of myeloid malignancies.

miRNA	Location		Expression	*In vitro* phenotype	*In vivo* phenotype	Bona fide targets
	Human	Mouse				
*miR-125*	Chr 19, 21, 11	Chr 17, 16, 9	• Highly expressed in HSCs • Reduced with differentiation	• Increases HSC self- renewal, decreases apoptosis, confers aberrant self-renewal in FL megakaryocyte erythroid progenitors (MEPs) and megakaryocytic progenitors (MPs)	• Enhances long-term reconstitution, reduces apoptosis in HSCs and progenitors, increases myeloid output at the expense of B cells. can lead to ALL at high doses as well as MPN/AML at very high doses	*Klf13, Bmf, Bak1, Dicer, ST18, ABTB1, CBFB*

*miR-142*	Chr 17	Chr 11	• Most highly expressed in lymphoid and myeloid progenitors • Downregulated in AML patient samples	• Ectopic expression in MLL-AF4+ cell lines suppressed cell proliferation, induced apoptosis, and downregulated multiple genes known to regulate self-renewal • Ectopic expression in in acute myeloid leukemia (AML) cell lines increases PMA– and ATRA-induced differentiation	Not investigated	*Cyclin T2, TAB2*

*miR-146*	Chr 5	Chr 11	• Expressed at relatively low levels in stem and progenitors and upregulated upon differentiation • Highly expressed in macrophages and T cells • Low expression in megakaryocytic progenitors (MPs)	• Overexpression leads to decreased megakaryopoiesis in human CD34+ HSPCs	• *miR-146-null* mice display hypersensitivity to LPS challenge, increased megakaryopoiesis. They also display pancytopenia and GM expansion with aging • *miR-146* KD in murine HSPCs followed by transplantation results in increased megakaryopoiesis, and an MDS phenotype with thrombocytosis and neutropenia	*TRAF6, IRAK1*

*miR-155*	Chr 21	Chr 16	• Relatively higher basal expression in HSPCs compared to more mature populations, such as erythroid progenitors • Induced in innate immune cells upon inflammatory stimuli as well as activated B and T cells	• *Ectopic expression of miR-155 in* K562 cells leads to a decreased CD41+ megakaryocytic differentiation	• Enforced expression in HSCs leads to development of a MPN/MPD with abnormal GM morphology, along with a reduction in erythroid progenitors in the bone marrow	*SHIP1, Cebp*β

*miR-29*	Chr 7, 1	Chr 6, 1	• Highest levels of expression in HSCs and MPPs, followed by LSCs, CMPs, MEPs, and GMPs; levels decrease with differentiation • Increased in patient LSCs, but not in non-LSCs	• Ectopic *miR-29* expression in AML cell lines leads to global DNA hypomethylation and reduces 5hmC levels; • Positively regulates myeloid differentiation, proliferation, and apoptosis	• Ectopic expression reduces the size of K562-laden tumors, via inhibiting proliferation and stimulating apoptosis and increases OS of mice with AML • Ectopic expression in early hematopoietic cells leads to induction of aberrant GMP self-renewal and the development of MPD with progression to AML	*DNMT3A, DNMT3B, Sp1, TET* family, *CDK6, CCNT2, AKT2, HBP*1

miRNAs with leukemogenic roles have been identified predominantly through high-throughput gene expression analyses. Despite the high number of miRNAs exhibiting altered expression in AML, sequencing of patient samples has revealed that somatic mutations in miRNAs are relatively rare events in human AML (Cancer Genome Atlas Research Network, 2013). Thus, miRNA deregulation in AML patients is largely due to alterations in transcriptional and/or post-transcriptional regulation. Furthermore, miRNAs are normally processed post-transcriptionally in a multi-step process involving DROSHA and DICER, each of which is regulated by several signaling molecules and RNA-binding proteins (Obernosterer et al., 2006; Thomson et al., 2006; Winter et al., 2009; Saj and Lai, 2011). Thus, understanding the complex regulation of the miRNAs that have been implicated in AML may provide novel avenues for therapeutic development. Moreover, many miRNAs are organized in clusters throughout the genome, and are expressed in a concerted manner. Paradoxically, however, individual members of a cluster can function in different or even opposite manners. For example, while *miR-125* can serve as an oncogene in the context of breast cancer (Iorio et al., 2005; Scott et al., 2007), its cluster partner, *let-7*, functions as a tumor suppressor in the same context (Lee and Dutta, 2007). These results suggest that miRNAs residing in clusters undergo different modes of post-transcriptional regulation. Thus, differing modes of post-transcriptional regulation of miRNAs within the same cluster might explain how such miRNAs serve opposing roles.

Although few miRNAs are mutated in human AML, many are known to be located in the proximity of breakpoints in chromosomal translocations/deletions (Calin et al., 2004). These observations suggest that miRNAs may contribute to disease pathogenesis, while the presence of additional genes in the deleted/translocated regions implies a potential cooperativity with miRNAs in leukemogenesis. In line with this idea, it is known that AML is a multi-step process, the understanding of which requires functional analyses of co-occurring disease alleles (Jan et al., 2012; Chen et al., 2014). This raises the possibility that genetic interaction studies between miRNAs and concurrently dysregulated or mutated genes might provide more accurate models to study disease. For instance, *miR-146a* is located in the del(5q) region observed in some MDS patients along with about 40 other genes, including the ribosomal protein RPS14 (Ebert et al., 2008). While *miR-146a* studies have provided precious insight into our understanding of MDS biology, they only partially recapitulate features of MDS. Thus, experiments employing approaches that evaluate loss-of-function del(5q) genes in combination with *miR-146a* loss might provide a more nuanced understanding of MDS pathogenesis.

Identification of target genes through which the pathogenic miRNAs in AML exert their effects is critical to understanding disease biology. A limited number of targets have been validated for each of these miRNAs and thus identification of *bona fide* target genes remains a major challenge in the field. Whereas some miRNAs mediate their function through a limited number of key target genes, others exert their action through many. To identify target genes mediating the effect of individual miRNAs, advanced target identification methods such as whole proteomics approaches and biochemical analyses of miRNA:mRNA interactions will be required. These techniques include RISC complex pull-down, RIP-Seq, HITS-CLIP and PAR-CLIP, which have been summarized in other reviews (Carroll et al., 2014). Furthermore, selective generation of knockin mouse models with mutations in the miRNA binding sites in the 3′-UTR of potential target genes (Jan et al., 2012) would assist the identification of relevant miRNA targets. Finding such target genes may provide additional promising therapeutic targets that have not yet been described (Garzon et al., 2010).

There are documented examples of the successful delivery of miRNA oligonucleotides to target tissues *in vivo* including the lung (Trang et al., 2011) and liver (Kota et al., 2009). Given the critical role of miRNAs in leukemogenesis, investigators have evaluated whether they might be effective therapeutic targets in AML. The potential use of miRNAs as therapeutic agents or targets in AML is underscored by their ability to regulate multiple signaling pathways that contribute to leukemogenesis (Li et al., 2007; Croce, 2008).

Two strategies may be employed to modulate miRNA activity: (i) enhancing the function of tumor supressor miRNAs using miRNA mimics, and (ii) inhibiting the function of oncogenic miRNAs by limiting their ability to bind to gene targets using antisense oligos or miRNA sponges (Garzon et al., 2010). For such therapies to find their way into the clinic, efficient and safe delivery of the targeting agents are required. Several groups have developed different delivery systems for miRNA targeting treatments (van Rooij and Kauppinen, 2014), including: (i) non-viral oligonucleotides; (ii) viral constructs overexpressing the miRNA or its antagomir (anti-sense oligo); and (iii) small-molecule delivery systems, where miRNA oligonucleotides are conjugated to small-molecules to ensure more efficient delivery (Ibrahim et al., 2011; Pramanik et al., 2011). Validating the optimal method of delivery will represent a significant challenge for miRNA-directed therapy in the future.

Among the leukemogenic miRNAs discussed in this review, *miR-155* and *miR-29* have been extensively studied as therapeutic targets in other contexts. Successful delivery of *miR-155* oligonucleotides to the bone marrow has been confirmed, with the inhibition of *miR-155* using a LNA (Takeshita et al., 2010) antagomir that was shown to alleviate symptoms of graft-versus-host disease (Ranganathan et al., 2012). In addition, systemically administered LNA antagonists against *miR-155* in a mouse inflammation model inhibited *miR-155*’s inflammatory effects in mouse splenocytes (Worm et al., 2009). In the context of AML, *miR-29b* oligonucleotide mimics appear to be promising therapies based on their effects on AML patient samples *in vitro* (Garzon et al., 2009b) and in K562-driven tumors *in vivo* (Garzon et al., 2009a). While *miR-29b* directed treatments show promise, these studies have not been performed using systemically delivered miRNA mimics. We expect to see such studies in the future aimed at identifying and validating more efficient and specific *in vivo* delivery systems for the treatment of AML.

## Conflict of Interest Statement

The authors declare that the research was conducted in the absence of any commercial or financial relationships that could be construed as a potential conflict of interest.
